# Association of MRI-derived radiomic biomarker with disease-free survival in patients with early-stage cervical cancer

**DOI:** 10.7150/thno.37429

**Published:** 2020-01-16

**Authors:** Jin Fang, Bin Zhang, Shuo Wang, Yan Jin, Fei Wang, Yingying Ding, Qiuying Chen, Liting Chen, Yueyue Li, Minmin Li, Zhuozhi Chen, Lizhi Liu, Zhenyu Liu, Jie Tian, Shuixing Zhang

**Affiliations:** 1 Medical Imaging Center, The First Affiliated Hospital of Jinan University, Guangzhou, 510630, China;; 2 Beijing Advanced Innovation Center for Big Data-Based Precision Medicine, School of Medicine, Beihang University, Beijing, 100191, China;; 3 CAS Key Laboratory of Molecular Imaging, Institute of Automation, Chinese Academy of Sciences, Beijing, 100190, China;; 4 Department of Radiology, the Third Affiliated Hospital of Kunming Medical University (Yunnan Cancer Hospital), Kunming, Yunnan, 650031, China;; 5 Sun Yat-sen University Cancer Center, State Key Laboratory of Oncology in South China, Collaborative Innovation Center for Cancer Medicine, Guangzhou, 510060, China;; 6 University of Chinese Academy of Sciences, Beijing, 100080, China.

**Keywords:** cervical cancer, magnetic resonance imaging, radiomics, disease-free survival

## Abstract

Pre-treatment survival prediction plays a key role in many diseases. We aimed to determine the prognostic value of pre-treatment Magnetic Resonance Imaging (MRI) based radiomic score for disease-free survival (DFS) in patients with early-stage (IB-IIA) cervical cancer.

**Methods:** A total of 248 patients with early-stage cervical cancer underwent radical hysterectomy were included from two institutions between January 1, 2011 and December 31, 2017, whose MR imaging data, clinicopathological data and DFS data were collected. Patients data were randomly divided into the training cohort (n = 166) and the validation cohort (n=82). Radiomic features were extracted from the pre-treatment T2-weighted (T2w) and contrast-enhanced T1-weighted (CET1w) MR imagings for each patient. Least absolute shrinkage and selection operator (LASSO) regression and Cox proportional hazard model were applied to construct radiomic score (Rad-score). According to the cutoff of Rad-score, patients were divided into low- and high- risk groups. Pearson's correlation and Kaplan-Meier analysis were used to evaluate the association of Rad-score with DFS. A combined model incorporating Rad-score, lymph node metastasis (LNM) and lymphovascular space invasion (LVI) by multivariate Cox proportional hazard model was constructed to estimate DFS individually.

**Results:** Higher Rad-scores were significantly associated with worse DFS in the training and validation cohorts (*P*<0.001 and* P*=0.011, respectively). The Rad-score demonstrated better prognostic performance in estimating DFS (C-index, 0.753; 95% CI: 0.696-0.805) than the clinicopathological features (C-index, 0.632; 95% CI: 0.567-0.700). However, the combined model showed no significant improvement (C-index, 0.714; 95%CI: 0.642-0.784).

**Conclusion:** The results demonstrated that MRI-derived Rad-score can be used as a prognostic biomarker for patients with early-stage (IB-IIA) cervical cancer, which can facilitate clinical decision-making.

## Introduction

As the fourth leading cause of cancer-derived death in women, cervical cancer is a global health problem 1, with an estimated 570,000 cases and 311,000 deaths worldwide in 2018 2. During the past decades, increasing early-stage cervical cancer has been detected largely due to the popularity of cancer screening 3, 4. For early-stage cervical cancer, the standard treatment is radical hysterectomy with pelvic lymph node dissection. However, locoregional recurrence or distant metastasis is not rare in patients with early-stage cervical cancer, only about 70% patients have a 5-year disease-free survival (DFS) 5. Prediction of patients' survival can help to determine whether more intensive observation and aggressive treatment regimens should be administered, which might improve clinical outcome.

Previous studies have identified the depth of invasion, lymph node metastasis (LNM) and lymphovascular invasion (LVI) as risk factors for recurrence and metastasis in cervical cancer patients 6, 7. These risk factors are determined by random sampling biopsy or surgery, which have limitations including procedure-related complications, sampling error, and interobserver variability 8. In this scenario, non-invasively prognostic biomarkers that allow assessment of tumor heterogeneity are warranted.

Radiomics has emerged as a promising method to evaluate tumor heterogeneity by extracting large set of high-dimensional features from a series of medical images, such as computed tomography (CT), positron emission tomography-computed tomography (PET/ CT) and magnetic resonance imaging (MRI) data 9. It has been conclusively shown that radiomic features could be used to diagnose precisely, evaluate treatment response, and predict survival in various types of cancers 10-15.

MRI is routinely used in clinical workup to diagnose, stage and monitor cervical cancer, with the advantages of lower cost and higher spatial and contrast resolution of pelvic tissues and organs, as well as no radiation 16-18. Nevertheless, whether the radiomic features extracted from MRI could be used to predict survival in patients with early-stage cervical cancer remains unclear.

Therefore, this study aimed to develop a radiomic score by pre-treatment MRI to estimate 3-year DFS in patients with early-stage cervical cancer, and to further construct a combined model incorporating the radiomic score and the clinicopathological features for the individual prediction of DFS.

## Methods

### Patients

Institutional ethics review board approval was acquired for this study, and written informed consent was not required for this retrospective study. This study was conducted in agreement with the Declaration Helsinki.

This retrospective study included patients with confirmed early-stage cervical cancer from two tertiary centers in a large metropolitan setting of China (Yunnan Cancer Hospital and Sun Yat-sen University Cancer Center) between January 1, 2011 and December 31, 2017. The patient demographics, laboratory test results, pretreatment MRI imaging data, pathologic results and survival outcome data were reviewed. All the patients met the following inclusion criteria: (a) patients with pathologically confirmed early-stage cervical cancer (Federation of Gynecology and Obstetrics [FIGO] stages IB-IIA); (b) patients underwent contrast-enhanced pelvic MRI scans within two-week period before surgery; and (c) patients underwent radical hysterectomy and bilateral pelvic lymph node dissection. The exclusion criteria of this study were as following: (1) patients treated with neoadjuvant chemotherapy or radiotherapy preoperatively; (2) patients with diagnosis of other cancers meanwhile; and (3) patients without clinical data including age, neutrophils, lymphocyte, platelet, squamous cell carcinoma antigen (SCCA) and human papillomavirus (HPV), pretreatment T2-weighted (T2w) and contrast-enhanced T1-weighted (CET1w) images. Finally, 248 patients (mean age of 47.77 ± 9.89 years) were included in this study. Supplementary Methods 2 showed the patient selection flowchart from the two centers. Eligible patients' data were randomly divided into a training cohort (n = 166) and an independent validation cohort (n = 82) at a ratio of 2:1.

### Treatments and follow-up

Radical hysterectomy and bilateral pelvic lymph node dissection were conducted for all patients. Adjuvant chemoradiotherapy after radical surgery was administered in 181 patients. Regular follow-up was conducted every 3-6 months during the first two years after operation, 2 times annually for 3-5 years, and then once a year thereafter. The endpoint of our study was DFS, which is defined as the period from the date of surgery to the date of the first locoregional recurrence, distant metastasis, death, or the last visit in follow up. Locoregional recurrences and distant metastasis were confirmed by gynecological examination, imaging modalities such as CT, MRI and positron emission tomography-computed tomography (PET/CT), or biopsy-proven. The available information was collected from patients' medical records.

### MR Image acquisitions

Abdomen and pelvic MRI examinations were conducted at least two weeks after biopsy to avoid the impact of post-biopsy inflammation and within two weeks before surgery. The MRI images were obtained by different MR devices at two institutions. To avoid the possibility of image information loss, we acquired the Digital Imaging and Communications in Medicine (DICOM) images from Picture Archiving and Communication System (PACS) directly without any compression or down sampling. Details regarding the acquisition parameters and MRI retrieval procedure were presented in Supplementary Methods 1.

### Radiomic analysis

**Figure 1:**
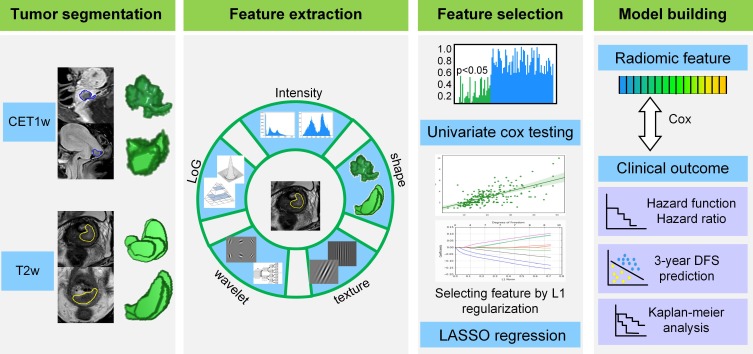
Radiomics framework of predicting the DFS of patients with cervical cancer. DFS: disease-free survival.

The radiomic analysis workflow included four steps as illustrated in Figure 1: tumor image segmentation, radiomic feature extraction, feature selection and model building.

### Tumor image segmentation

We used open-source ITK-SNAP software (www.itksnap.org) for three-dimensional manual segmentation in axial T2w and sagittal CET1w images. Regions-of-interest (ROIs) were manually delineated by a radiologist who had 8 years of experience in gynecological MR imaging interpretation, and validated by a senior radiologist with 10 years of experience in segmentation results validation.

### Radiomic feature extraction

After manually segmenting the tumor ROI, we standardized the T2w and CET1w images by z-score normalization to obtain a standard normal distribution of the image intensities. This procedure was aimed at reducing the image intensity shift caused by different equipments and scanning parameters. Afterward, we resampled CET1w and T2w images into 1 mm × 1 mm and 0.65 mm × 0.65 mm, respectively. Here, 1 mm × 1 mm and 0.65 mm × 0.65 mm were the median voxel spacing of CET1w and T2w images in this dataset. Then we extracted radiomic features from T2w and CET1w images respectively through an open-source software PyRadiomics 19. Considering the relatively low resolution and large variance between different equipments for axial MR images, we extracted two dimensional (2D) features in axial T2w images and sagittal CET1w images instead of three dimensional (3D) features to increase the robustness of features. Specifically, we extracted 2D features from all the 2D slices of tumor ROI, and averaged the feature values in all the image slices as the final results for each patient 20-21.

For each slice, we extracted 1299 2D radiomic features including: i) intensity features (n = 19): these features were the first-order statistics calculated from the tumor intensities such as entropy, reflecting the signal intensity of different tumors, ii) shape features (n = 16): these features represented the size and shape information of tumors, which showed prognostic value in cervical cancer, iii) texture features (n = 74): these features measured the relationship between each tumor voxel and its surrounding environments, which can quantify intra-tumor heterogeneity and the use of complex tumor patterns such as size-zone matrix, iv) wavelet features (n = 736): we decomposed MR images into low and high frequencies and extracted the features in group i and ii from each frequency range. The wavelet transformation enabled us to quantify high-dimensional multi-frequency tumor information that is difficult to be visually interpreted, and v) Laplacian of Gaussian (LoG) features (n = 454): these features were textural features extracted through a Laplacian of Gaussian spatial band-pass filter. These features described the tumor information from multi-scale space that combined both the very detailed and macroscopic tumor texture patterns.

### Radiomic feature selection

Although the radiomic features reflected tumor information from various perspectives, not all of them were associated with the DFS in cervical cancer. Consequently, we used a two-step feature selection method to retain only the most strong features that are significantly associated with DFS. First, we evaluated the predictive performance of each radiomic feature by univariate Cox regression in the training cohort. Specifically, we built a univariate Cox proportional hazards model (Cox model) for each radiomic feature in the training cohort, and calculated the P value of the feature in predicting DFS. Afterward, the features with P value less than 0.05 were treated as significant prognostic factors and selected as candidate features. Second, we used regularized multivariate logistic regression with the LASSO penalty for multivariate feature selection. The LASSO regularization involved a parameter λ to control the number of selected features where a larger λ retains more features. To obtain an optimal feature number and avoid over-fitting, we used 5-fold cross-validation in the training cohort to choose the optimal λ. The λ value that maximized the C-index in the training cohort was selected as the optimal regularization parameter, and the feature number was therefore determined automatically by the λ.

### Creation of clinicopathological model for DFS prediction

We included 7 candidate clinicopathological findings which showed potential prognostic values to build the clinicopathological model, including age, histological type, differentiation grade, HPV, SCCA level, LNM and LVI status. Among the clinicopathological findings, we firstly used the univariate Cox proportional hazards model to select the significant prognostic factors in the training cohort. Then, significant variables in the univariate Cox model (*P* <0.05) were included in the multivariate Cox model to build a clinicopathological model for DFS prediction. Detailed calculating process of the Cox model and the Rad-score were provided in Supplementary Methods 3. The performance of the model was evaluated by C-index. The C-index indicates the concordance between the model-predicted DFS and the actual DFS on all the patients, where a C-index around 0.5 means poor predictive value and a C-index around 0.7 indicates good predictive value.

### Development and validation of radiomic score and combined model on DFS prediction

In the training cohort, we used a multivariate Cox proportional hazard model to predict a radiomic score (Rad-score) indicating the relative disease progression hazard for each patient. For a given patient, the Cox model used the radiomic feature of this patient to generate a Rad-score larger than zero. A small Rad-score means that the disease progression risk is relatively low, and the DFS for this patient is consequently long; a large Rad-score means high-risk of disease progression, and a relatively short DFS. As described in Supplementary Methods 3, the Cox model used an exponential combination of the selected radiomic feature to generate the Rad-score.

To further evaluate whether the clinicopathological findings can improve the performance of the Rad-score, we used the Rad-score and the significant clinicopathological features to build a combined multivariable Cox model (combined model) for DFS prediction. The prognostic performance of the Rad-score and the combined model were also evaluated by C-index.

### 3-year DFS probability prediction of various models

Considering the Cox model is capable of predicting the DFS probability at a given time point, we also used the Cox model to estimate 3-year DFS. Receiver-operating characteristic (ROC) analyses were performed to estimate the prognostic performance of the three models in predicting 3-year DFS.

### Statistical Analysis

All the statistical analyses in this study were performed with SPSS 21 and python 2.7. The t test or Mann-Whitney U test of independent samples were conducted to assess the significance of age, neutrophils, lymphocyte, platelet, Histological type, HPV and SCCA level between the training cohort and the validation cohort. The Chi-squared test was conducted to evaluate the significance of the categorical variables such as FIGO stage, differentiation grade, LNM and LVI between the training and validation cohorts. The LASSO-based feature selection and Cox proportional hazards model building were implemented using “scikit-learn” and “lifelines” package. Kaplan-Meier curve was analyzed using the "rms" package and Log-rank test. A two-tailed *P*-value less than 0.05 was considered statistically significant.

## Results

### Patient characteristics

A total of 248 patients were included from two cohorts. The patient characteristics were presented in Table 1. The mean age of patients was 47.77 ± 9.89 years. The median follow-up time was 30 months (range, 6-96 months). The results demonstrated that there was no significant difference between the training cohort and the validation cohort (*P* = 0.151-0.990).

### DFS prediction performance of the radiomic score

A total of 18 radiomic features were selected for radiomic score building (Supplementary Table S1, Figure S1). In the training cohort, the radiomic score showed good performance on DFS prediction (C-index, 0.786; 95% CI: 0.753-0.820). In the validation cohort, the performance of the radiomic score was further confirmed (C-index, 0.753; 95% CI: 0.696-0.805). The hazard ratio (HR) for radiomic score was 2.259 (95%CI: 2.124-2.394) in the training cohort.

### Kaplan-Meier analysis of radiomic score

**Figure 2:**
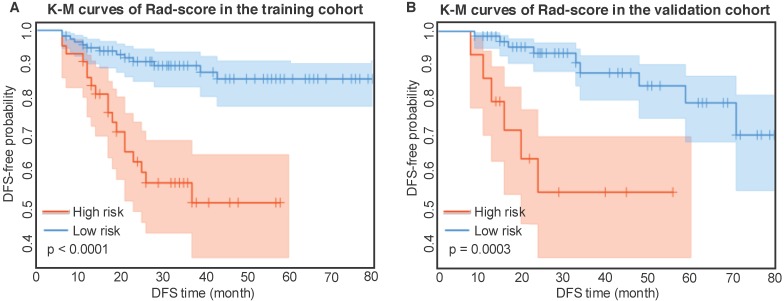
Kaplan-Meier analysis of the Rad-score. A) Kaplan-Meier curves of the Rad-score in the training cohort. Vertical lines indicate censored data. Shadows represent 95% CI. B) Kaplan-Meier curves of the Rad-score in the validation cohort. DFS: disease-free survival. CI: confidence interval.

According to the Rad-score, we further divided patients into high-risk and low-risk groups, and performed Kaplan-Meier analysis to validate the prognostic value of the Rad-score. We used the mean hazard score of the training cohort as a cut-off value to divide patients into high-risk and low-risk groups. As shown in Figure 2, higher Rad-scores were significantly associated with worse DFS in the training cohort and the validation cohort (both with *P* <0.001). Figure 4 showed two representative patients with distinctly different DFS time (14 months vs 64 months). Although they had almost the same clinicopathological features, their Rad-scores (2.046 vs 0.237) were significantly different.

### Performance and validation of the combined model on DFS prediction

Only two clinical features (LNM and LVI) were selected to create a clinicopathological model. This model achieved a poor performance in DFS estimation, with a C-index of 0.711 (95% CI: 0.671-0.753) in the training cohort and 0.632 (95%CI: 0.567-0.700) in the validation cohort. The combined model incorporating the radiomic score and the two clinicopathological features showed improvement in the training cohort (C-index, 0.813; 95%CI: 0.780-0.845), but showed no improvement in the validation cohort (C-index, 0.714; 95%CI: 0.642-0.784) when compared with radiomic score.

### 3-year DFS probability prediction of various models

**Figure 3:**
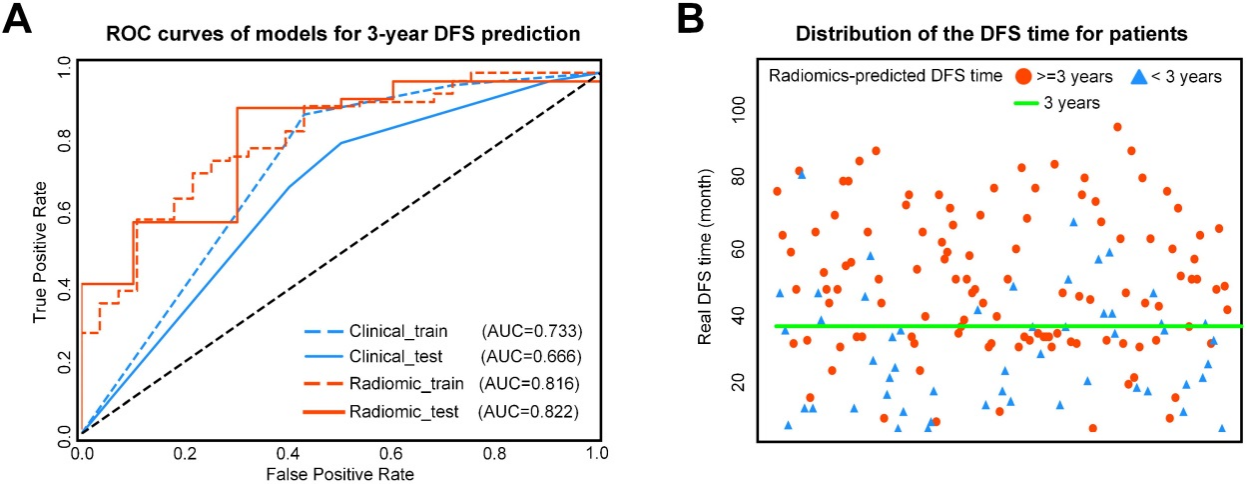
A) ROC curves of the two models for 3-year DFS probability prediction. B) Distribution of the DFS time for patients. The red circle represents patients who are predicted to have DFS time longer than 3 years by the Rad-score, and the blue triangle represents patients who are predicted to have DFS time less than 3 years by the Rad-score. Most patients who are predicted to have DFS timelonger than 3 years distribute above the patients who are predicted to have DFS time less than 3 years. ROC: receiver-operating characteristic; DFS: disease-free survival.

**Figure 4:**
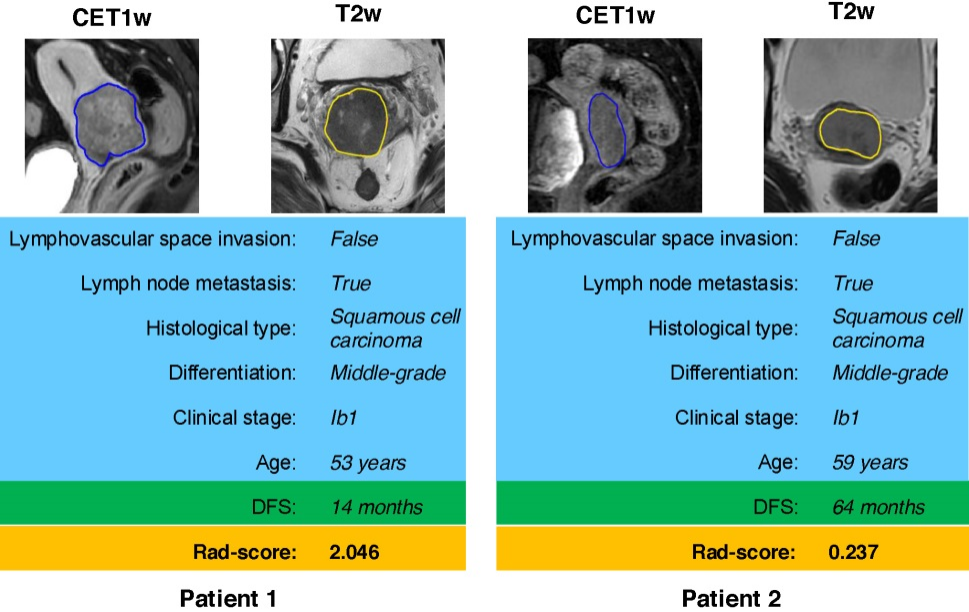
MR images of two patients with significantly different DFS time. Although patient 1 and patient 2 have similar clinicopathological characteristics, their Rad-score are different and discriminative. DFS: disease-free survival.

For 3-year DFS probability prediction, the clinicopathological model achieved an area under the receiver operating characteristic curve(AUC) of 0.666 (95%CI: 0.595-0.742), sensitivity of 0.805 (95%CI: 0.760-0.857), specificity of 0.500 (95%CI: 0.372, 0.635), and accuracy of 0.745 (95%CI: 0.700-0.796) (Table 2, Figure 3). The radiomic score yielded an AUC of 0.822 (95%CI: 0.765-0.882), sensitivity of 0.780 (95%CI: 0.729-0.833), specificity of 0.700 (95%CI: 0.583-0.817), and accuracy of 0.765 (95%CI: 0.718-0.813) (Table 2, Figure 3). The Rad-score showed significant difference between patients with DFS time >3 years and <3 years (*P*< 0.0001 in the training cohort;* P* = 0.0010 in the validation cohort). In Supplementary Table S2, we also provided the performance of the Rad-score on predicting DFS at multiple time points.

The combined model yielded an AUC of 0.759 (95% CI: 0.678-0.843), sensitivity of 0.780 (95%CI: 0.730-0.835), specificity of 0.700 (95%CI: 0.581-0.822), and accuracy of 0.765 (95% CI: 0.717-0.817) (Table 2, Figure 3). Therefore, the combined radiomic model showed no performance improvement in 3-year DFS estimation when compared with the Rad-score.

## Discussion

In our study, we evaluated the prognostic value of MR-derived radiomic features on patients with IB-IIA cervical cancer treated by radical hysterectomy. The results showed that LASSO-Cox based radiomic score had favorable predictive performance in DFS estimation. Our study would help to determine whether more intensive observation and aggressive treatment regimens should be administered in patients with worse DFS, with the aim of assisting clinical treatment and healthcare decisions.

Radiomics provided underlying diagnostic, therapeutic, and prognostic information by noninvasively extracting useful imaging features from medical images 22. Some previous studies applied PET radiomics to predict survival of cervical cancer, which was more accurate than conventional clinical factors 23-25. However, these studies had smaller sample size and did not focus on early-stage cervical cancer treated by surgery. Their findings were of limited clinical relevance because the treatment patients received was a strong indicator for survival. However, Brooks FJ et al. showed that radiomic features based on PET could not provide additional information for small tumor lesions due to the limited spatial resolution of PET imaging with the large voxel size sampling 26. The value of MRI radiomic model has been proven in predicting the LNM and LVI status in patients with cervical cancer preoperatively 27, 28. Giving this background, radiomic features based on MRI was available to predict the survival of early-stage cervical cancer patients.

The radiomic score that combined the T2w and CET1w images yielded a C-index of 0.753 (95% CI: 0.696-0.805) on DFS prediction and an AUC of 0.822 (95% CI: 0.765-0.882) on 3-year DFS prediction in the validation cohort, which were higher than either clinical model or the combined model. This indicated that the radiomic score may already contain information in clinical factors, and can mine more prognostic information than clinical factors by observing the whole tumor scope and extracting high-dimensional features (e.g., wavelet and LoG features). Thus, it could be used as a surrogate biomarker to improve the prognostic ability pretreatment. Meanwhile, the Rad-score could stratify patients into high-risk and low-risk groups. Patients with higher Rad-scores have the worse DFS, which suggested that some low-risk patients would have received unnecessary radical hysterectomy treatment; whereas for patients with high risk of recurrence and metastasis, a systemic adjuvant treatment would be more beneficial. Our findings would initiate a pivotal step to enable surgeons to tailor treatment basing on the specific clinical and radiomic features for high-risk and low-risk patients with early-stage cervical cancer.

Of the 1299 radiomic features, 18 were identified to be predictive for DFS, which includes 10 features derived from CET1w and 8 features extracted from T2w images. This may indicate that CET1w images probably contains more prognostic information than T2w images. Importantly, shape flatness was included in the 10 CET1w- derived features. This feature characterized the shape of tumor: small flatness value indicates an irregular tumor shape. In the radiomic score, a tumor with small flatness generates a poor prognostic outcome, which is in line with a prior study that tumor sphericity is a poor prognostic marker for breast cancer 11. In addition, eight wavelet features and one LoG feature were selected from the CET1w images. The wavelet features reflected tumor information in eight space domains, and the LoG features reflected tumor information from three frequency domains. This result demonstrated that the raw CET1w images may include limited prognostic features; however, through a wavelet and LoG transform of the raw CET1w images, much prognostic information can be mined. This further reflected the advantages of radiomics method since it is good at mine high-dimensional information that is difficult to be sensed manually. For example, the “SumEntropy” in a wavelet subspace and the “Skewness” in a LoG subspace were selected, indicating that in a high-dimensional wavelet and LoG spaces, the tumor heterogeneity described by entropy and the intensity of tumor have prognostic value. Similarly, one intensity feature, two texture features, one wavelet feature and four LoG features were selected from the T2w images.

In a previous study, pretreatment HPV genotype was reported as a prognostic biomarker in cervical cancer 29, 30. So far, the correlation between the HPV level and survival time in cervical cancer patients is seldom studied and remains unknown. The result of our data showed that there is no significant correlation between them. The potential reason for the unconformity result is that the HPV test involves all of the risk HPV genotypes, with no specific categories, whereas some high-risk HPV genotypes are not associated with the prognosis of cervical cancer 31. Our study demonstrated that LVI and LNM were identified as significant independent prognostic factors for DFS, which is consistent with previous studies 32-34. The mean DFS is 24.75 months for patients with LVI, and 38.56 months for patients without LVI (*P*< 0.001). Similarly, the mean DFS is 29.91 months for patients with LNM, and 37.50 for patients without LNM (*P* = 0.009). In this study, the clinical stage (IB-IIA) was not associated with DFS; the possible reason is that the survival of patients in early-stage is relatively good.

Despite the favorable results of the radiomic score, our study also has some limitations. First, although the number of patients in this study was large as compared with previous studies, larger and prospective datasets would be needed to optimize the performance of the model in the future. In addition, since the DFS of patients in the two institutions was different; therefore we combined the data from two institutions when dividing the training and validation cohorts. In future work, we hope to perform external validation in a different center. Second, because of the multicenter scanner settings, the MR images may be affected by different scanners and protocols. Therefore, we combined images from multiple scanners and tried to eliminate the device-effect by image standardization and robust 2D feature extraction. However, a more comprehensive method to balance the scanner-variance worth future exploring. Third, we mined 18 prognostic features from MR image and compared their performance with clinical factors. However, the association between the radiomic feature and biological level events was not explained. In the future, we will explain the radiomic features at biological level by combining gene profile. In addition, deep learning as an emerging method in medical image analysis may provide valuable features that are complementary to radiomic features^35^. Due to limited training samples, directly using deep learning in this study can probably cause overfitting; however, combining deep learning features with the selected radiomic features worth future exploring. For example, we can use transfer learning to extract deep learning features without the need for large training data, and then combine the deep learning features with the selected radiomic features. This strategy can combine the advantages of both deep learning and hand-crafted radiomic features.

In conclusion, this study provides a noninvasive and pretreatment prognostic biomarker for the DFS of cervical cancer based on MRI. Moreover, for each cervical cancer patient, the radiomic score can predict the hazard risk of the patient being disease-free and stratify the patient into high-risk and low-risk groups. The study may offer some important insights into precise treatment, providing valuable guidance for clinical physicians regarding the treatment strategies including radical hysterectomy or chemoradiation on early-stage cervical cancer patients.

## Supplementary Material

Supplementary figures and tables.

## Figures and Tables

**Table 1:** Comparison of clinical characteristics of patients between the training and validation cohorts

Characteristics	Training cohort (n=166)	Validation cohort (n=82)	p
**Age (years, mean ± SD)**	47.69 ±9.46	47.93 ±10.77	0.864
**Neutrophils (10-9/L, mean ± SD)**	4.77 ± 2.79	6.23 ±9.38	0.151
**Lymphocyte (10-9/L, mean ± SD)**	2.06 ± 4.13	1.84±1.05	0.545
**Platelet (10-9/L, mean ± SD)**	264.5 ±82.54	270.7 ±87.26	0.542
**SCC (ng/mL)**	4.84 ±7.51	5.48 ±8.09	0.237
**HPV**	535.53 ±764.31	601.87 ±847.68	0.236
**Histological type (%)**			0.669
Squamous cell carcinoma	141 (84.94%)	68 (82.93%)	
Adenocarcinoma	22 (13.25%)	11 (13.41%)	
Adenosquamous carcinoma	1 (0.60%)	2 (2.44%)	
Small cell carcinoma	2 (1.20%)	1 (1.22%)	
**FIGO stage (%)**			0.712
IB	110 (66.27%)	57 (69.51%)	
IIA	56 (33.73%)	25 (30.49%)	
**Differentiation (%)**			0.400
Low grade	100 (60.24%)	46 (56.10%)	
Middle grade	64 (38.55%)	33 (40.24%)	
High grade	2 (1.20%)	3 (3.66%)	
**Lymph node metastasis (%)**			>0.990
Non-metastasis	131 (78.92%)	64 (78.05%)	
Metastasis	35 (21.08%)	18 (21.95%)	
**Lymphovascular space invasion (LVI, %)**			0.452
Non-LVI	132 (79.52%)	61 (74.39%)	
LVI	34 (20.48%)	21 (25.61%)	
**Mean DFS time (months, mean ± std)**	34.97 ±20.44	35.79 ±21.38	0.747

**Table 2:** Model performance on predicting DFS and 3-year DFS probability

Models	Cohorts	C-Index (95% CI)	AUC (95% CI)	ACC (95% CI)	Sensitivity (95% CI)	Specificity (95% CI)
**Clinical model**	training	0.711 (0.671, 0.753)	0.733 (0.689, 0.773)	0.754 (0.723, 0.785)	0.802 (0.769, 0.836)	0.607 (0.530, 0.678)
	validation	0.632 (0.567, 0.700)	0.666 (0.595, 0.742)	0.745 (0.700, 0.796)	0.805 (0.760, 0.857)	0.500 (0.372, 0.635)
**Radiomic score**	training	0.786 (0.753, 0.820)	0.816 (0.779, 0.854)	0.746 (0.713, 0.780)	0.756 (0.718, 0.794)	0.714 (0.649, 0.784)
	validation	0.753 (0.696, 0.805)	0.822 (0.765, 0.882)	0.765 (0.718, 0.813)	0.780 (0.729, 0.833)	0.700 (0.583, 0.817)
**Combined model**	training	0.813 (0.780, 0.845)	0.849 (0.816, 0.880)	0.754 (0.722, 0.786)	0.756 (0.719, 0.795)	0.750 (0.679, 0.811)
	validation	0.714 (0.642, 0.784)	0.759 (0.678, 0.843)	0.765 (0.717, 0.817)	0.780 (0.730, 0.835)	0.700 (0.581, 0.822)

Note: CI represents confidence interval. C-Index represents Harrell's concordance index, which measures the performance of the DFS prediction. AUC represents area under the receiver operating characteristic curve, and ACC is accuracy. AUC and ACC evaluate the performance of the 3-year DFS prediction.

## Abbreviations

MRI: Magnetic Resonance Imaging
DFS: disease-free survival
LASSO: Least absolute shrinkage and selection operator
LNM: lymph node metastasis
LVI: lymph-vascular invasion
CT: computed tomography
PET/CT: positron emission tomography-computed tomography
T2w: T2-weighted
CET1w: contrast-enhanced T1-weighted
DICOM: Digital Imaging and Communications in Medicine
PACS: Picture Archiving and Communication System
ROI: Regions-of-interest
LoG: Laplacian of Gaussian
HPV: human papillomavirus
SCCA: squamous cell carcinoma antigen
ROC: Receiver-operating characteristic
AUC: Area under the receiver operating characteristic curve
