# Supplementary material for: Association of MRI-derived radiomic biomarker with disease-free survival in patients with early-stage cervical cancer
Source: Theranostics. 2020 Jan 16;10(5):2284–92. doi: 10.7150/thno.37429 (PMC7019161; doi:10.7150/thno.37429)
Supplement: Supplementary file 1 — Supplementary figures and tables. [file thnov10p2284s1.pdf]

Supplementary Material

Supplementary Methods 1. Magnetic resonance image acquisition parameters used in the present study.

All the patients from the Yunnan Tumor Hospital were examined using the SIEMENS 1.5T Avanto MRI with the following scanning parameters: axial T2-weighted spin-echo images (repetition time [TR]/ echo time [TE]: 4000/100 ms, field of view [FOV] = 20 × 18 cm, number of excitation [NEX] = 4, slice thickness = 3 mm, spacing between slices = 0.3 mm) and sagittal contrast- enhanced T1-weighted spin-echo images (TR/TE: 4.65/1.55 ms, FOV = 26 × 22 cm, NEX = 8, slice thickness = 3.6 mm, spacing between slices = 0.7 mm).

The patients from Sun Yat-sen University Cancer Center were examined using the 1.5T GE Signa MRI and the 3.0T GE Discovery750 MRI.

The 3.0T GE Discovery750 MRI acquisition parameters were as follows: axial T2-weighted spin-echo images (TR/TE: 3966/86 ms, FOV = 36 × 36 cm, NEX = 2, slice thickness = 5 mm, spacing between slices = 1mm) and sagittal contrast- enhanced T1-weighted spin-echo images (TR/TE: 5.25/1.82 ms, FOV = 28× 28 cm, NEX = 1, slice thickness = 3 mm, spacing between slices = 0 mm).

The 1.5T GE Signa MRI acquisition parameters were as follows: axial T2-weighted spin-echo images (TR/TE: 3283/87 ms, FOV = 36 × 36 cm, NEX = 2, slice thickness =5 mm, spacing between slices =1mm) and sagittal contrast- enhanced T1-weighted spin-echo images (TR/TE: 4.1/1.96 ms, FOV = 28× 26 cm, NEX = 1, slice thickness = 3 mm, spacing between slices = 0 mm).

The image matrices were 512 × 512 for all the patients.

Supplementary Methods 2. Patients flowchart

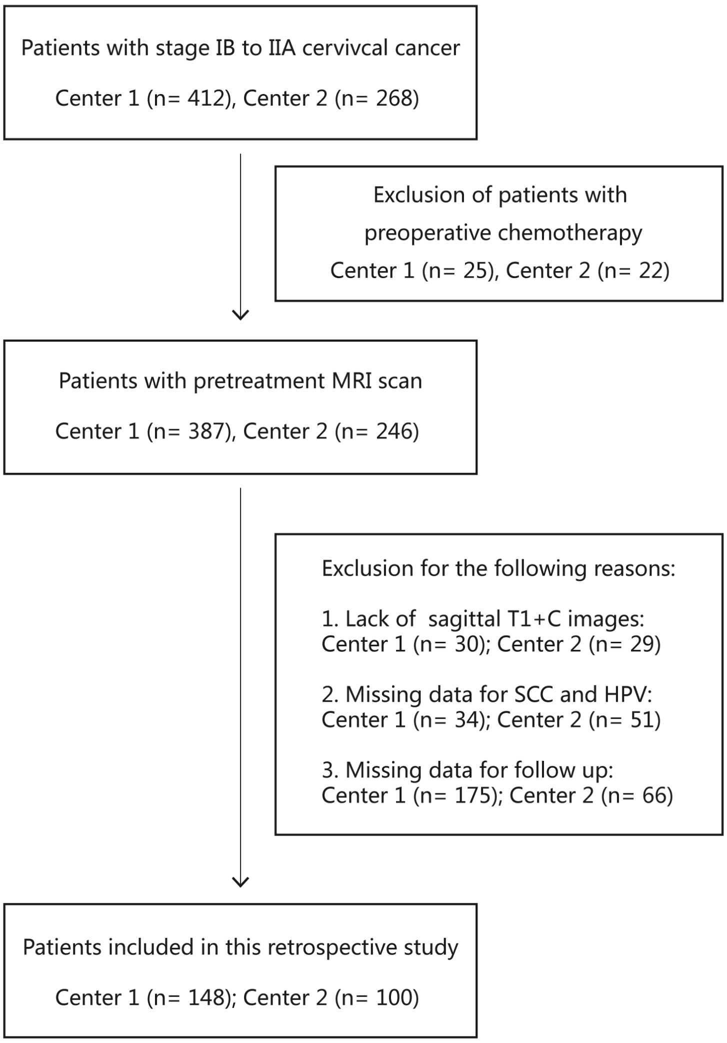

Supplementary Methods 3. Mathematical description of the Cox model

The Cox proportional hazard model is defined as follows [1]:

$$\mathrm{h}(t|X) = h_0(t)e^{\beta^T X} \quad \text{Eq. 1}$$

where $\mathrm{h}(t|X)$ is the hazard ratio of patient X at time t, $h_0(t)$ is the baseline hazard that is independent of patient, $X = [x_1 \quad x_2 \quad \cdots \quad x_n]^T$ is the radiomic feature for the patient, and $\beta = [\beta_1 \quad \beta_2 \quad \cdots \quad \beta_n]^T$ is a vector of coefficients for the radiomic feature X, which is determined in the training phase.

The baseline hazard $h_0(t)$ is the same for each patient (i.e., independent of patient). To achieve personalized DFS prediction, we mainly focus on the $e^{\beta^T X}$ in $\mathrm{h}(t|X)$ since this part varies from person to person. Consequently, we simplify $\mathrm{h}(t|X) = h_0(t)e^{\beta^T X}$ into $\mathrm{h}(X) = e^{\beta^T X}$. Here, $\mathrm{h}(X)$ is defined as the hazard ratio for the patient X, which is also defined as the Rad-score in this study. This value is independent of time, and the range of this value is larger than 0, indicating the relative risk or hazard of the patient X being disease progression.

The computational process was implemented in lifelines package in Python (https://lifelines.readthedocs.io/en/latest/index.html).

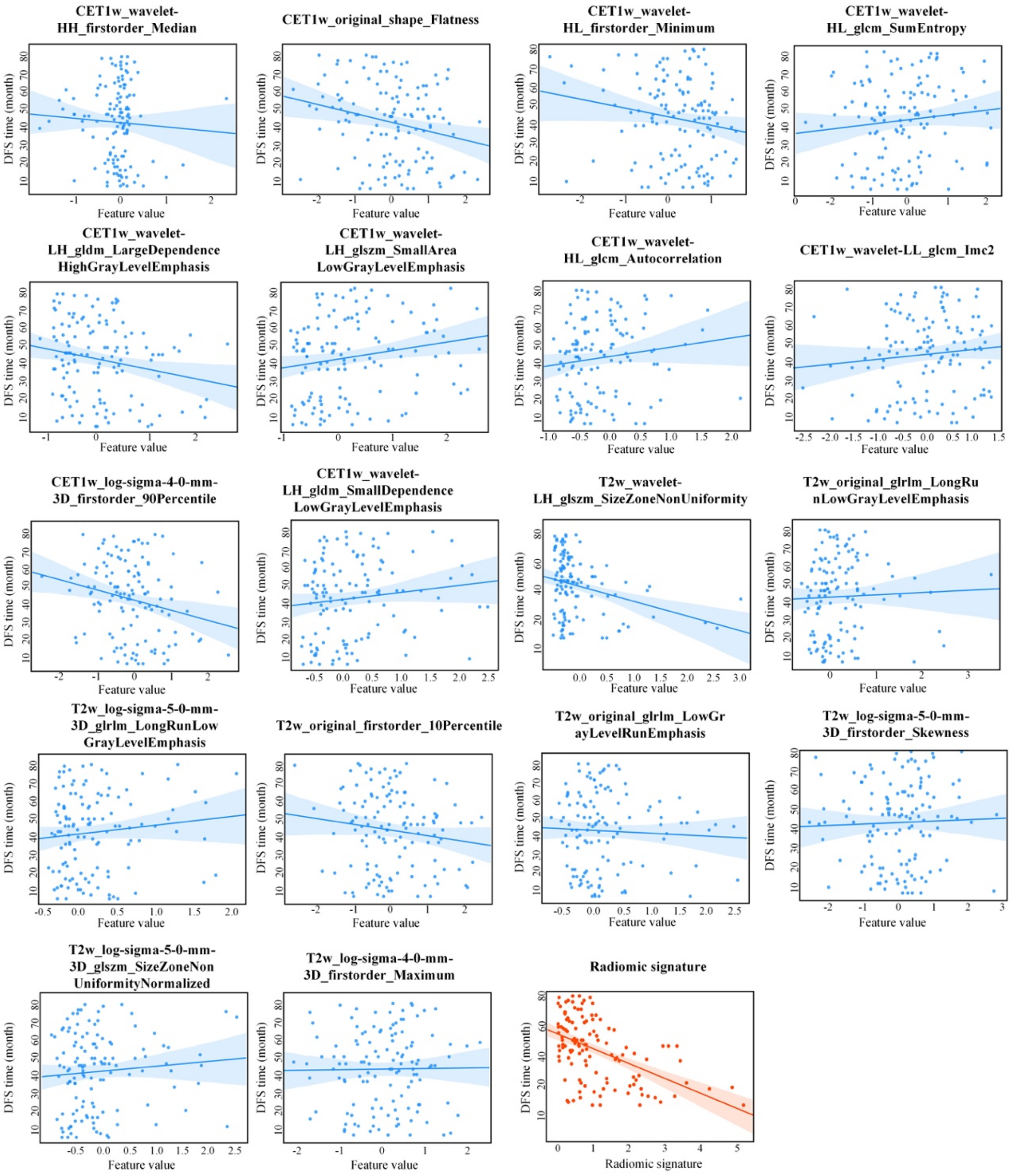

Supplementary Figure S1. Distribution of the selected radiomic features and the Rad-score. The line in each figure is the linear regression of the points, and the shadow represents confidence interval.

Supplementary table S1. The name of the 18 selected radiomic feature

	Feature name
feature 1	CET1w_wavelet-HH_firstorder_Median
feature 2	CET1w_original_shape_Flatness
feature 3	CET1w_wavelet-HL_firstorder_Minimum
feature 4	CET1w_wavelet-HL_glcm_SumEntropy
feature 5	CET1w_wavelet-LH_gldm_LargeDependenceHighGrayLevelEmphasis
feature 6	CET1w_wavelet-LH_glszm_SmallAreaLowGrayLevelEmphasis
feature 7	CET1w_wavelet-HL_glcm_Autocorrelation
feature 8	CET1w_wavelet-LL_glcm_Imc2
feature 9	CET1w_log-sigma-4-0-mm-3D_firstorder_90Percentile
feature 10	CET1w_wavelet-LH_gldm_SmallDependenceLowGrayLevelEmphasis
feature 11	T2w_wavelet-LH_glszm_SizeZoneNonUniformity
feature 12	T2w_original_glrlm_LongRunLowGrayLevelEmphasis
feature 13	T2w_log-sigma-5-0-mm-3D_glrlm_LongRunLowGrayLevelEmphasis
feature 14	T2w_original_firstorder_10Percentile

feature 15	T2w_original_glrlm_LowGrayLevelRunEmphasis
feature 16	T2w_log-sigma-5-0-mm-3D_firstorder_Skewness
feature 17	T2w_log-sigma-5-0-mm-3D_glszm_SizeZoneNonUniformityNormalized
feature 18	T2w_log-sigma-4-0-mm-3D_firstorder_Maximum

Note: CET1w represents the radiomic feature is extracted from CET1w images, and T2w represents the radiomic feature is extracted from T2w images.

Supplementary Table S2. Performance of the Rad-score on predicting DFS at multiple time points.

Time	cohort	AUC	ACC
2-year	training	0.799 (0.760, 0.840)	0.738 (0.709, 0.770)
	validation	0.812 (0.748, 0.873)	0.815 (0.777, 0.854)
2.5-year	training	0.814 (0.774, 0.851)	0.750 (0.711, 0.784)
	validation	0.737 (0.669, 0.812)	0.791 (0.743, 0.844)
3-year	training	0.816 (0.781, 0.852)	0.737 (0.705, 0.771)
	validation	0.822 (0.765, 0.886)	0.765 (0.719, 0.812)
3.5-year	training	0.847 (0.813, 0.884)	0.759 (0.723, 0.799)
	validation	0.759 (0.690, 0.827)	0.675 (0.617, 0.733)
4-year	training	0.864 (0.829, 0.898)	0.771 (0.731, 0.810)
	validation	0.764 (0.690, 0.836)	0.657 (0.592, 0.721)
